# Facile Attachment of TAT Peptide on Gold Monolayer Protected Clusters: Synthesis and Characterization

**DOI:** 10.3390/nano5031211

**Published:** 2015-07-21

**Authors:** Ndabenhle M. Sosibo, Frankline K. Keter, Amanda Skepu, Robert T. Tshikhudo, Neerish Revaprasadu

**Affiliations:** 1 Advanced Materials Division, DST/Mintek Nanotechnology Innovation Centre, Mintek, Private Bag X3015, Randburg 2125, South Africa; E-Mails: neerishrev@gmail.com (N.M.S.); franklinek@mintek.co.za (F.K.K.); amandas@mintek.co.za (A.S.); 2 Department of Chemistry, University of Zululand, Private Bag X1001, KwaDlangezwa 3886, South Africa

**Keywords:** nanoparticles, MPCs, gold, TAT, peptide, biotin

## Abstract

High affinity thiolate-based polymeric capping ligands are known to impart stability onto nanosized gold nanoparticles. Due to the stable gold-sulfur bond, the ligand forms a protective layer around the gold core and subsequently controls the physicochemical properties of the resultant nanogold mononuclear protected clusters (AuMPCs). The choice of ligands to use as surfactants for AuMPCs largely depends on the desired degree of hydrophilicity and biocompatibility of the MPCs, normally dictated by the intended application. Subsequent surface modification of AuMPCs allows further conjugation of additional biomolecules yielding bilayer or multilayered clusters suitable for bioanalytical applications ranging from targeted drug delivery to diagnostics. In this study, we discuss our recent laboratory findings on a simple route for the introduction of Trans-Activator of Transcription (TAT) peptide onto the surface of biotin-derivatised gold MPCs via the biotin-strepavidin interaction. By changing the surface loading of biotin, controlled amounts of TAT could be attached. This bioconjugate system is very attractive as a carrier in intercellular delivery of various delivery cargoes such as antibodies, proteins and oligonucleotides.

## 1. Introduction

The application of nano-sized inorganic materials in chemical processes, therapeutics and diagnostics, among others, has generated a multidisciplinary effort in the design and fabrication of smart nanomaterials [1,2,3]. The versatility of gold nanoparticles depends on their physico-chemical properties that can be fine-tuned in order to improve their electronic, diagnostic and therapeutic functions and performances [4,5,6]. The sensitivity of the surface plasmon resonance (SPR) of these materials to their immediate environment renders them as responsive optical detection tools, which can be used for the detection of biological and chemical agents [7,8,9,10,11]. Thus, there is great interest in this class of materials.

The synthesis of these nanomaterials requires considerable control of their composition, size, shape, stability and dispersion properties owing to applications in various scientific fields. The exploitability of gold nanoparticles in terms of their physical, chemical, electronic and optical properties hinges on the synthetic design approaches. These systems can be further functionalized to form such systems as monolayer protected clusters (MPCs) [12,13] and conjugates carrying important cargoes such as proteins, antibodies, enzymes and other biomolecules. MPCs are applied in the development of therapeutic systems for drug delivery, bio-labeling and diagnostics for point-of-care devices. As such, interesting work on the development of PEGylated (polyethylene glycol) gold clusters have been reported over the last two decades [14,15,16]. The MPCs were shown to have extreme stability in high electrolyte media and are known to impart hydrophilicity, stability and low nonspecific binding due to the PEG aliphatic chain.

Protocols for the preparation of stable hydrophobic gold and silver MPCs have been developed over the years [12]; however, these MPCs are insoluble in water and limits their applications, especially in medicine since they are incompatible with biomolecules and biological *milieu*. Pioneering work by Murray and co-workers [17], who introduced water soluble ligands onto the gold nanoparticle surface, paved the way for the development of robust multifunctional MPCs containing specific targeting ligands that are essential in addressing various biological applications. This was achieved through the incorporation of polyethylene glycol (PEG)-based ligands that imparts water solubility, biocompatibility and lower non-specific interactions of these materials with biomolecules [17]. As such, there have been a lot of conjugates developed. Perhaps the most studied are the gold MPCs with biological molecules such as proteins, nucleic acids, and DNA attached to them. These materials have found use in areas such as bio-imaging, detection and are promising drug delivery vehicles amongst other applications [18,19]. The immobilization of biomolecules onto the inorganic surfaces has been achieved through various synthetic techniques, namely (i) electrostatic (charge) attachment [20,21,22]; (ii) specific affinity immobilisation [23,24,25]; and (iii) covalent attachment [26,27]. The choice of the immobilization route is generally based on the type of biomolecule and whether it is in its native or fusion form.

Here, we report the introduction of Trans-Activator of Transcription (TAT) peptide *via* the biotin-streptavidin interactions. TAT is a small basic cell-penetrating peptide possessing a generic sequence, YGRKKRRQRRR, although the C- and N- termini have been extended using a wide range of sequences or coupled with other moieties [28]. TAT peptide is a short arginine-rich protein transduction domain, which has found applications in the delivery of biologically active therapeutic biomolecules, namely peptides, proteins and antisense oligonucleotides [29,30,31]. Although the mechanism of internalization of TAT has not been determined conclusively, it is believed that a lipid raft-dependent macropinocytosis is most likely the method of entry [32]. This specialized form of fluid phase endocytosis is both temperature- and energy-dependent [33]. Previous work by De La Fuente *et al.* [34] encompassed the introduction of TAT peptide onto CdS nanoparticles *via* the carbodiimide coupling reaction. This approach involved the formation of an amide bond between the amine moiety of the TAT and the carboxyl functional group of the PEG ligands attached to the nanoparticles.

In this work, we present an alternative facile route to attach biotinylated TAT peptide onto the surface of streptavidin layered gold clusters. This approach is simple as the risk of disrupting the peptide functionality is minimized by using the amino acid residues as anchoring points. The risk of introducing toxic reagents during the coupling reaction, thereby requiring rigorous purification methods, is also avoided. Moreover, the peptide content can be varied by adjusting the fractional ratios of the ligands binding the target streptavidin on the surface of the gold MPCs. The resultant TAT-derivatized conjugates are attractive tools in the areas of drug delivery.

## 2. Results and Discussion

### 2.1. Synthesis of 14 nm Citrate-Capped Gold Nanoparticles, AuNPs

**Figure 1:**
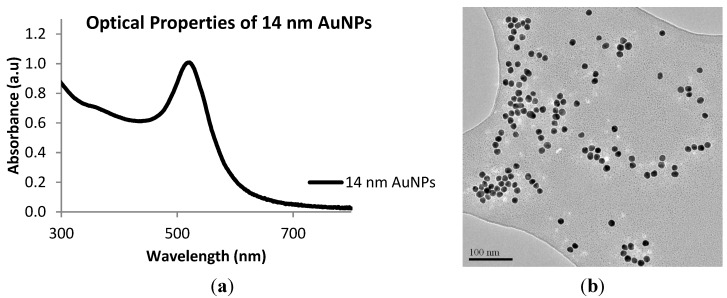
(**a**) UV-vis spectrum of 14 nm AuNPs and (**b**) Transmission Electron Microscopy (TEM) image (100 nm scale) of 14 nm AuNPs.

The starting material, 14 nm gold nanoparticles, was prepared using established methodologies [35,36]. Briefly, the 14 nm AuNPs were prepared by the citrate reduction method and their optical properties and their morphological characteristics determined by UV-vis spectroscopy and microscopy (TEM). The UV-vis spectrum of AuNPs is shown in Figure 1a with an SPR peak appearing at 520 nm. A representative transmission micrograph is shown in Figure 1b. These results are in agreement with the literature results [37]. The zeta (ζ-) potential recorded for the AuNPs was −35.5 mV, indicating that the particles are stable post production.

**Scheme 1:**
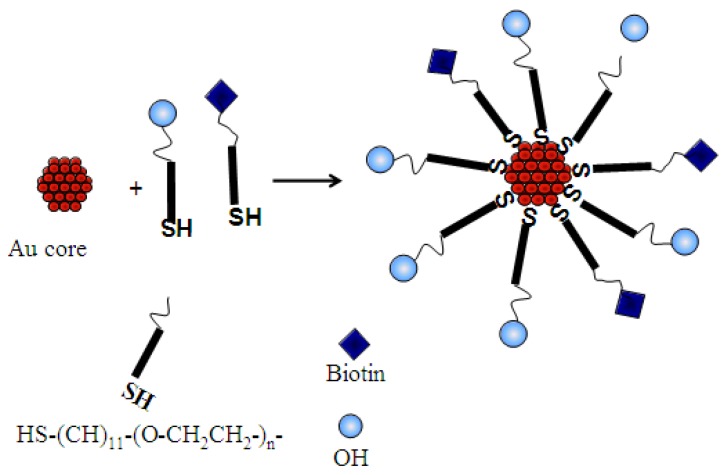
PEGylation of the gold nanoparticles with biotinylated polyethylene glycol (PEG) ligand.

Subsequently, the 14 nm AuNPs were stabilized with polyethylene glycol (PEG) molecules, PEG-OH (468.69 g/mol) and PEG-biotin (694.00 g/mol), in a process called PEGylation. In this study, we discovered that the use of 100% PEG functionalized biotin to stabilize AuNPs was not successful as the nanoparticles agglomerated within a short time. To solve this problem, the co-stabilization method was employed by using two PEG molecules. PEG-OH was chosen because of its versatility with regards to solubility and biological compatibility. As such, the new AuMPCs were prepared by using a mixture of PEG-biotin: PEG-OH in a 1:10 ratio as shown in Scheme 1. A quick flocculation test of the resultant AuMPCs, using 5 M NaCl, showed that the particles were quite stable over time.

**Figure 2:**
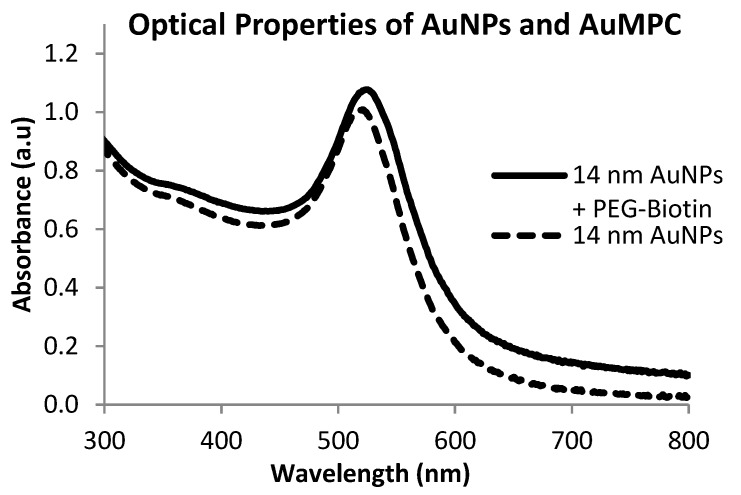
UV-vis spectrum of 14 nm AuNPs and PEG-biotin functionalised mononuclear protected clusters (MPCs).

From the optical absorption spectrum (Figure 2), there was no much shift in the SPR peak of the MPCs (recorded at 524 nm) compared to the citrate-capped particles (starting material) at 522 nm.

The slight red shift in the SPR peak is possibly due to ligand contributions, albeit insignificant. Notably, this increase in red-shift observed was not accompanied by peak broadening signifying the retention of the dispersity of the MPCs. This is an indication that the integrity of the gold nanoparticles was maintained upon introduction of PEG molecules onto the surface of AuNPs.

**Figure 3:**
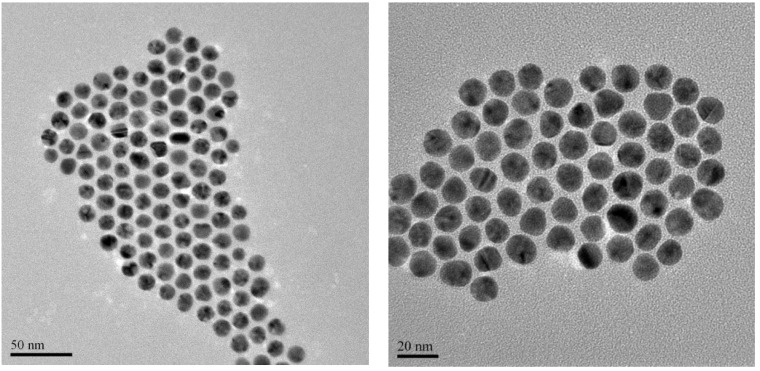
TEM images of PEG-biotin functionalised MPCs at 20 and 50 nm scale.

TEM measurements showed no aggregation of particles (Figure 3). The micrograph shows orderly formations with almost equidistant arrangement, and confirms the monodispersity of the AuMPC. This observation was in good agreement with the optical absorption observations.

### 2.2. Biofunctionalization with Streptavidin

The introduction of streptavidin on the surface of gold MPCs was expected to be robust because of high affinity biotin-streptavidin interactions. Streptavidin binds four moles of biotin per one mole of the protein corresponding to 16.5–18 mg of biotin bound per gram of streptavidin [38]. This interaction is an essential biorecognition tool for protein-ligand interactions with a large dissociation constant of ≈10^−^^14^ M, one of the largest non-covalent ligand-protein interactions in the literature [21]. One of the advantages of this system is the low isoelectric point (pI) of streptavidin, which reduces non-specific binding of streptavidin at common working pH ranges [39]. Since the biotin on the gold MPCs surface is presented in a long chain alkylated PEG, the “buried” streptavidin binding sites can easily be accessed [40].

**Scheme 2:**
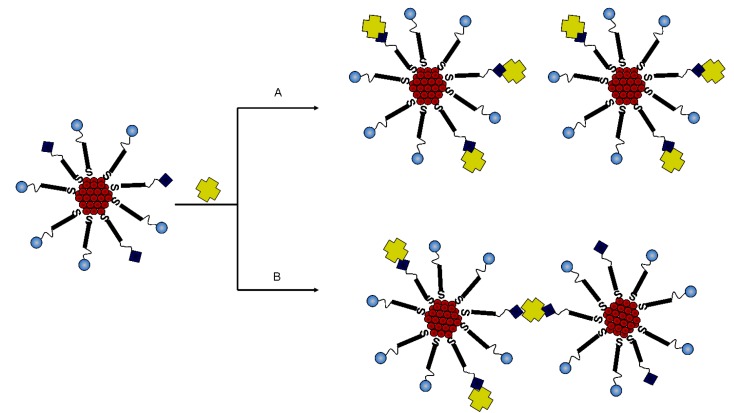
Possible modes of interaction between the biotin-containing MPCs with streptavidin. (**A**) Excess streptavidin will allow the saturation of all the biotin molecules on the surface of the gold whereas; (**B**) lack of surplus streptavidin could lead to separate nanoparticles being interconnected via the streptavidin molecules and thus aggregation.

**Figure 4:**
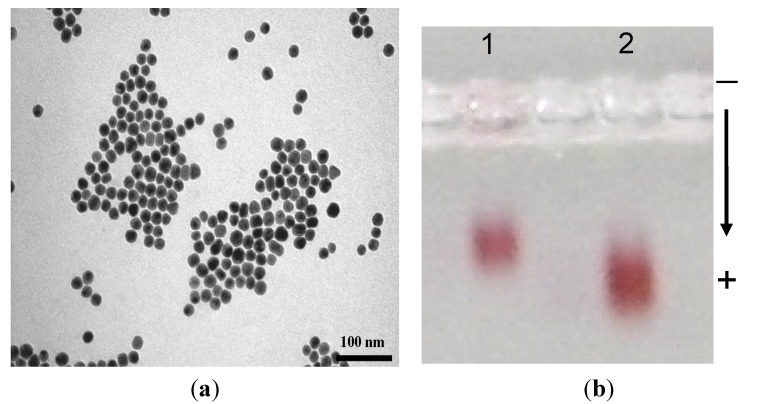
(**a**) The TEM micrograph of the streptavidin gold conjugates (at 100 nm scale) and (**b**) agarose gel electrophoresis measurements of streptavidin gold bioconjugates, showing the mobilities of lane 1: streptavidin gold conjugates and lane 2: PEG-biotin AuMPC.

The most favoured binding is shown in Scheme 2A. However, it is also possible for the streptavidin to cause aggregation by way of one streptavidin connecting two adjacent molecules (Scheme 2B). The optical measurements of AuMPC showed a single narrow peak at around 525 nm. Furthermore, the TEM measurements confirmed that the particles were monodispersed (Figure 4a), invariably ruling out the occurrence of path B (Scheme 2).

Agarose gel electrophoresis (Figure 4b) was conducted on the streptavidin gold conjugates and compared with the starting PEG-biotin gold MPC. Since the electrophoretic mobilities are dependent on charge, size and isoelectric point of the target biomolecules, there was a distinct change in mobility of the two sets of nanomaterials with the PEG-biotin AuMPC migrating faster that the streptavidin-linked gold conjugate. This result was a clear indication that the immobilisation of the streptavidin was successful.

### 2.3. Immobilization of Biotinylated TAT Peptide

**Scheme 3:**
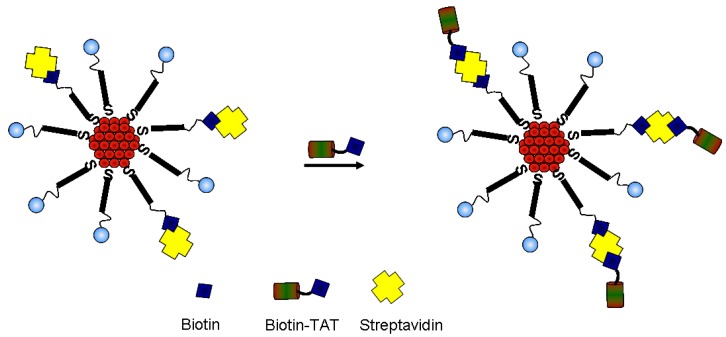
A schematic representation of the introduction of Trans-Activator of Transcription (TAT) peptide onto the streptavidin gold conjugates.

Since the tetrameric streptavidin binds up to four molecules of biotin, further biotinylation on the surface of the conjugates is possible as illustrated in Scheme 3.

**Figure 5:**
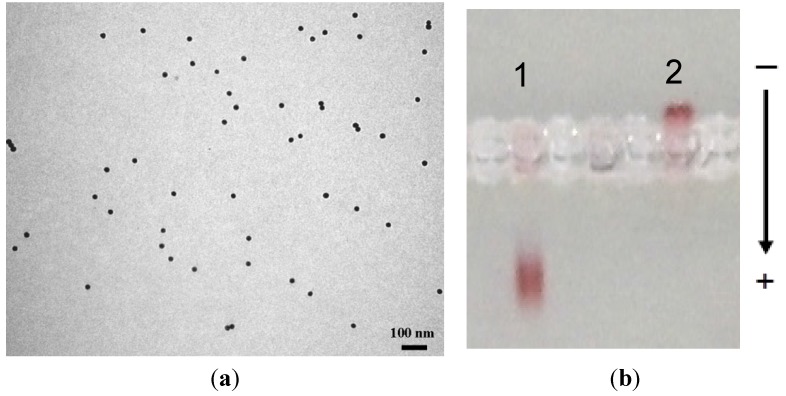
(**a**) TEM micrograph of the TAT-biotin gold conjugates and (**b**) agarose gel electrophoresis measurements of TAT gold bioconjugates, showing the mobilities of lane 1: streptavidin gold conjugates and lane 2: TAT peptide gold conjugates.

The biotinylated TAT peptide was introduced on the surface of the streptavidin conjugates. Similar to the introduction of streptavidin, an excess of the peptide was used. The excess peptide was then removed by way of centrifuging the particles at 10,000 rpm and the nanoconjugate re-suspended in borate buffer. The UV-vis measurements gave a single sharp SPR peak signifying that the integrity of the nanoconjugate was mantained. This was further corroborated by agarose gel electrophoresis conducted that showed a marked difference in the migration of the gold conjugate compared to the AuMPC precursor (Figure 5a). Whereas the AuMPC precursor migrated to the anode, gold conjugate built from the AuMPC did not migrate. TAT peptide is a highly hydrophic considering the arginine and lysine residues in the sequence; thus, it is likely for it to have near neutral charge, and it is not surprising that it did not migrate in the gel. Once again, the TEM measurements confirmed the monodispersity of the nanoparticles (Figure 5b). The gel data coupled with TEM data showed that TAT peptide was successfully attached via the biotin-streptavidin interactions. The synthetic route offers a direct immobilization of the peptide without the need to utilise the amine groups of the amino acid residues in the peptide. Furthermore, the TAT conjugates were stable even at high salt concentration (5 M NaCl) and can be redispersed in water and borate buffer.

Mathematical calculations were used to quantify the amount of molecules attached to the surface of the gold nanoparticles. The concentration of the 14 nm gold nanoparticles used was 1.2 × 10^14^ nps/mL. A total of PEG-OH and PEG-biotin was calculated to be 5.50 × 10^17^ molecules. This translates to 4583 PEG molecules (4294 PEG-OH/289 PEG-biotin) per nanoparticle, which gives a density of ~7 PEG molecules/nm^2^. This is comparable to those reported in literature such as, the packing density of 7.8 molecules/nm^2^ and 4.3 molecules/nm^2^ for 5–100 nm gold nanoparticles conjugated with 3-mercaptopropionic acid and mercapto-(PEG)_4_-carboxylic respectively [40,41,42,43]. Although streptavidin and TAT-biotin were used in excess, the pre-calculated ratios was such that PEG-biotin:streptavidin:TAT-biotin was 1:1:1 meaning that streptavidin and TAT-biotin molecules attached were of the same number as the PEG-biotin (289 molecules). Notably, streptavidin and TAT biotin are anchored on the PEG-biotin and not directly to the surface of the gold nanoparticles.

## 3. Experimental Section

### 3.1. Materials, Instrumentation and Methods

The HAuCl_4_.3H_2_, trisodium citrate (Na_3_C_6_H_5_O_7_·2H_2_O), Caliber™ glycerol, sephadex G-25 (diameter = 20–50 m), sephacryl 100-HR, phosphate buffered saline, Tris borate-EDTA buffer (dry blend) were all purchased from Sigma Aldrich while Agarose SFR™ was purchased from AMRESCO and TAT-biotin from AnaSpec Inc. The PEG molecules, 2-{2-[2-(2-{2-[2-(1-mercaptoundec-11-yloxy)-ethoxy]-ethoxy}-ethoxy)-ethoxy]-ethoxy}-ethanol (PEG-OH, C23H48O7S, 468.69 g/mol) and N-(2-{2-[2-(2-{2-[2-(1-mercaptoundec-11-yloxy)-ethoxy]-ethoxy}-ethoxy)-ethoxy]-ethoxy}-ethyl) biotinamide (PEG-biotin, C_33_H_63_N_3_O_8_S_2_, 694.00 g/mol) were purchased from Prochimia Surfaces. All the chemicals were used without further purification. High purity double-distilled water was used in all the experiments.

The UV-Visible spectroscopy was performed on a PerkinElmer Lambda UV-Vis Spectrophotometer (PerkinElmer, Waltham, MA, USA) with 1 cm quartz cuvettes in the 200–1000 nm wavelength range. The samples were further characterized on a Phillips CM120 Biotwin transmission electron microscope (TEM; Phillips, Amsterdam, Netherlands) equipped with an EDAX detector and Gatan crystorage and operated at 120 kV. The specimens were prepared by dropping a dilute solution of the sample containing the nanoparticles on a carbon-coated copper grid (400 mesh) and allowing the solvent to dry out at room temperature. The images were captured using the embedded self-imaging system Megaview III digital camera (GmbH, Munchen, Germany). The surface charge of the nanoparticles was determined by way of zeta potential measurements carried out on a Zetasizer Nano ZS from Malvern Instruments (Malvern, UK).

Centrifugation was used to purify the nanoparticles. This was achieved by using the Hettich MIKRO 22R cooling centrifuge (Andreas Hettich GmbH & Co. KG, Tuttlingen, Germany), where the samples were generally centrifuged in 1.5 mL Eppendorf tubes at 12,000 rpm. Agarose gel electrophoresis experiments were conducted using the Centipede™ Wide Gel Electrophoresis System. Typically, the AuMPCs were supplemented with 1% glycerol (10% *v*/*v* of the total AuMPCs volume) and loaded at 10 µL per well of 1% agarose gel. The experiments were run in 1X TBE (0.89M Tris, 0.89M Boric Acid, 0.002M EDTA) at 60 V constant voltage for 0.5–2 h.

### 3.2. Synthesis

#### 3.2.1. Synthesis of Citrate-Capped Gold Nanoparticles, AuNPs

The 14 nm citrate-capped particles were prepared using the previously reported methodologies [35,36]. The resultant citrate-capped gold nanoparticles were of average size 14 ± 2 nm.

#### 3.2.2. Synthesis of PEG-Biotin Monolayer Protected Clusters, AuMPCs

The MPCs containing 1% PEG-biotin were synthesised and co-stabilised with PEG-OH (mass to mass ratios). Into 20 mL of citrate-capped gold nanoparticles (2 nM), methanolic solutions of PEG-biotin (8.4 mg/mL, 4.8 µL) and PEG-OH (18.8 mg/mL, 21.3 µL) were mixed and added simultaneously. The reaction mixture was stirred for a further 3 h and the resultant 1% PEG-biotin gold MPCs were centrifuged (12,000 rpm, 20 min, 22 °C) and washed three times with high purity water. The purified gold MPCs were stored in a 2 mL polypropylene tube until further use.

### 3.3. Biofunctionalization with Streptavidin

Following the synthesis and purification of 1% PEG-biotin-functionalized gold MPCs, streptavidin was introduced via the biotin-avidin (BA) interaction. An aqueous solution of streptavidin (1 mg/mL, 25 µL) was introduced into the concentrated PEG-biotin gold MPCs (800 µL, 16 nM). The volume was made up to 2 mL by adding PBS buffer (0.01 M phosphate buffer, 0.0027 M KCl and 0.137 M NaCl). The reaction mixture was mixed thoroughly by swirling followed by incubation in the refrigerator at 4 °C for 48 h. Excess streptavidin was washed three times with PBS by centrifugation three times (12,000 rpm, 20 min, 22 °C). The streptavidin gold conjugates were stored in PBS at 4 °C.

### 3.4. Biomolecular Immobilization with Biotin-TAT

The immobilization of the biotinylated cell-penetrating peptide (CPP), TAT was conducted on the streptavidin conjugates. Into the streptavidin conjugates (850 µL, 1.5 nM), a PBS (0.01 M phosphate buffer, 0.0027 M KCl and 0.137 M NaCl) solution of biotin-TAT (20 µL) was added. The mixture was gently swirled to mix and then incubated at 4 °C for 48 h to complete the reaction. The resultant TAT gold conjugates were washed of excess peptide with PBS through centrifugation (12,000 rpm, 20 min, 22 °C). The resultant conjugates were stored in PBS at 4 °C until further use.

## 4. Conclusions

The synthesis and subsequent biomolecular functionalization of gold nanoparticles was performed with relative ease. The introduction of bulky PEG molecules prevents aggregation of the nanoparticles. From our results, it is clear that streptavidin is a versatile option for linking the PEGylated biotin to the biotinylated TAT peptide. The same approach can be used to design multilayered nanoconjugates for various applications. In this study, the introduction of the TAT peptide on the gold nanoparticles represents a tool kit that is important in areas in biolabeling whereupon the microscopically visible gold core can be visualised.
